# Comparative Genomic Analysis of 130 Bacteriophages Infecting Bacteria in the Genus *Pseudomonas*

**DOI:** 10.3389/fmicb.2018.01456

**Published:** 2018-07-04

**Authors:** Anh D. Ha, Dee R. Denver

**Affiliations:** Department of Integrative Biology, Oregon State University, Corvallis, OR, United States

**Keywords:** bacteriophage, biodiversity, genomics, natural selection, *Pseudomonas*

## Abstract

Bacteria of the genus *Pseudomonas* are genetically diverse and ubiquitous in the environment. Like other bacteria, those of the genus *Pseudomonas* are susceptible to bacteriophages which can significantly affect their host in many ways, ranging from cell lysis to major changes in morphology and virulence. Insights into phage genomes, evolution, and functional relationships with their hosts have the potential to contribute to a broader understanding of *Pseudomonas* biology, and the development of novel phage therapy strategies. Here we provide a broad-based comparative and evolutionary analysis of 130 complete *Pseudomonas* phage genome sequences available in online databases. We discovered extensive variation in genome size (ranging from 3 to 316 kb), G + C percentage (ranging from 37 to 66%), and overall gene content (ranging from 81–96% of genome space). Based on overall nucleotide similarity and the numbers of shared gene products, 100 out of 130 genome sequences were grouped into 12 different clusters; 30 were characterized as singletons, which do not have close relationships with other phage genomes. For 5/12 clusters, constituent phage members originated from two or more different *Pseudomonas* host species, suggesting that phage in these clusters can traverse bacterial species boundaries. An analysis of CRISPR spacers in *Pseudomonas* bacterial genome sequences supported this finding. Substantial diversity was revealed in analyses of phage gene families; out of 4,462 total families, the largest had only 39 members and there were 2,992 families with only one member. An evolutionary analysis of 72 phage gene families, based on patterns of nucleotide diversity at non-synonymous and synonymous sites, revealed strong and consistent signals for purifying selection. Our study revealed highly diverse and dynamic *Pseudomonas* phage genomes, and evidence for a dominant role of purifying selection in shaping the evolution of genes encoded in them.

## Introduction

Bacteriophages are the most abundant, dynamic and genetically diverse forms of life in the biosphere, with an estimate existence of 10^31^ phages worldwide, continuously infecting 10^23^ to 10^24^ bacterial cells every second (Hendrix, 2010; Keen et al., 2017). That is, if we were to lay all 10^31^ phage particles in the world side by side, the length of that line would be about 200 million light years [estimated by Domingo et al. (2008)], or about 1.892 × 10^21^ kilometers. The paramount number and diversity of phages, which exceed those of bacteria, are reflected in their varied habitats, reproduction cycles, infection strategies, and ability to shift hosts. Investigations of phage genomes by sequencing and metagenomics have already suggested enormous genomic diversity, complexity, and mosaic evolution (Lawrence et al., 2002), but a detailed understanding of phage genetic diversity and evolution remains a challenge.

Bacteria of the genus *Pseudomonas* are Gram-negative, aerobic gammaproteobacteria with more than 200 species identified (Özen and Ussery, 2012). *Pseudomonas* bacteria live in a wide variety of ecological habitats: water, soil, and in associations with fungi, plants, animals, and human. Members of the genus include the opportunistic human pathogen *P. aeruginosa*, well-known as the leading cause of pneumonia and acute nosocomial infections, which poses a serious challenge to human patients with burn wounds, cancer, and cystic fibrosis (Iglewski, 1996). *P. syringae*, with more than 50 identified pathovars preying on diverse plant species, is a major and very well-studied plant pathogen (Benson et al., 2007; Morris et al., 2008). Alternatively, *P. fluorescens* and *P. chlororaphis* are called ‘Plant Growth Promoting Rhizobacteria’ (PGPR) for their ability to protect plants against pathogens by competitive colonization (Capdevila et al., 2004), production of antifungal compounds and antibiotics (Raaijmakers et al., 2010; Calderón et al., 2015), and alteration of auxin level which benefits plant growth (Kang et al., 2006). Other notable members of this bacterial genus include *P. tolaasii* - the cause of brown blotch in cultivated mushrooms, the equine pathogen *P. mallei*, and the soil inoculant *P. putida*. Altogether, *Pseudomonas* bacteria have highly important and diverse environmental, biological, and human health-related impacts.

*Pseudomonas* phages play key roles in shaping the diversity and evolution of bacteria through many mechanisms (Paulsen et al., 2005), including horizontal gene transfers and alteration of host virulence (Hayashi et al., 1990; Sano et al., 2004). Research of phages has been applied broadly in epidemiological studies of *Pseudomonas* diseases over the last 50 years, including the identification and sub-classification of pathogenic strains (Bernstein-Ziv et al., 1973; Lindberg and Latta, 1974; Bergan, 1978), DNA transduction to introduce genes to bacterial cells (Darzins and Casadaban, 1989; Rolain et al., 2011), and therapeutic strategy for antibiotic-resistant *Pseudomonas* infections (Hraiech et al., 2015; Pires et al., 2015; Yayan et al., 2015). Therefore, improved understanding of *Pseudomonas* phages should contribute to a broader understanding of *Pseudomonas* bacteria and to the development of molecular tools and clinical applications.

Whole genomic sequencing of phage virions and prophages, facilitated by the modern advance in sequencing technology and metagenomics, has been offering an exciting new avenue for understanding phages and their impacts on host bacteria. To date, approximately 8,300 complete bacteriophage genomes have been sequenced, and about 400 were isolated from members of *Pseudomonas*, providing valuable source for investigation into the diversity and complexity of *Pseudomonas-*infecting phages. Whole-genome comparative analysis has been successfully applied in previous studies focused on mycobacteriophages (Hatfull et al., 2010), *Staphylococcus aureus* phages (Kwan et al., 2005), *Bacillus* phages (Grose et al., 2014), and *Enterobacteriaceae* phages (Grose and Casjens, 2014), the findings of which all highlighted the remarkable dynamics and diversity of phages. In each study, a large percentage of total genes identified (e.g., 47.2% of mycobacteriophage genes and 58% of *Bacillus* phage genes) were unique, lacking any clear discernible relationships to other genes. Phage genomes were commonly grouped into clusters of related phages, with outliers characterized as ‘singletons’ that lacked strong relationships with other genomes in the analysis. By identifying clusters of closely related genomes, whole-genome analysis provides the opportunity to evaluate the diversity and complex relationship between phages, as well as exploring their dynamic host range.

Comparative analysis of phage sequences also offers an avenue to understanding selection pressures acting on phage genes, providing further insight into their history of evolution. A common population genetic metric to study the evolutionary process of protein-coding genes is the ratio of nucleotide diversity at non-synonymous and synonymous sites (π_N_ and π_S_, respectively). In coding regions, nucleotide changes between sequences may either lead to amino acid substitutions (non-synonymous) or keep the protein sequences unchanged (synonymous). Assuming that synonymous sites evolve neutrally and represent ‘background’ mutational variation in genome, the ratio π_N_/π_S_ provides insights into the direction and magnitude of natural selection pressure. The ratio π_N_/π_S_ < 1 is considered a signature of purifying selection, which maintains genetic stability of the genes of interest. It is hypothesized that the majority of phage protein coding genes, especially those encoding structural functions and survival benefits, are under purifying selection (Domingo et al., 2008; Hartl, 2014), therefore have π_N_/π_S_< 1. Positive selection is inferred when π_N_/π_S_> 1. If nucleotide substitutions happen randomly with respect to protein-coding function, the sequences of interest are likely evolving under neutrality and have π_N_/π_S_ = 1.

To further study the deep divergence and potential for host range evolution of phages infecting *Pseudomonas*, we examined genome statistics, putative gene contents, and performed whole-genome comparative analysis of 130 phage sequences available in NCBI GenBank databases. To probe into phage gene evolution, we reported, for the first time, the pattern of selection acting on phage putative gene orthologs by comparing the nucleotide diversity patterns π_N_/π_S_ in predicted ORF families. Our study has contributed new insights into the diversity and evolution of phages infecting *Pseudomonas* and facilitate comparison with phages infecting other bacteria.

## Materials and Methods

### Phage Genome Sequences

One hundred and thirty complete DNA genomes then available on the NCBI GenBank database were downloaded as fasta sequences on February 15, 2016. Only entries with the description “complete genome” in the “Definition” field and the isolation source “*Pseudomonas*” at the genus level, stated in the “host” field, were included in downstream analyses. The GenBank accession numbers of these genomes were included in **Supplementary Table S1**. It was stated in the GenBank entries, of the 130 phages selected for study, that they were isolated from six different host species: *P. chlororaphis, P. tolaasii, P. syringae, P. putida, P. fluorescens*, and *P. aeruginosa*, from varied sources (i.e., water sewage, hospital wastewater, and environmental samples) at different geographical locations.

### Genomic Analysis

#### Genome Annotation

All 130 phage genomes analyzed in this study were re-annotated to ensure annotation uniformity. Gene prediction was performed with GeneMark.hmm 3.26 (Besemer and Borodovsky, 1999; Zhu et al., 2010) using the heuristic parameters, Glimmer 3.02 (Salzberg et al., 1998; Delcher et al., 1999), and BLASTN when necessary, followed by genome annotation by Phage Rapid Annotation using Subsystem Technology (RAST) (Aziz et al., 2008; Overbeek et al., 2014; Brettin et al., 2015) and PHASTER web server (Zhou et al., 2011; Arndt et al., 2016). Gene annotation was not manually curated systematically in all genomes. While annotation using the gene calling programs GeneMarkS and Glimmer were previously shown to be accurate and suitable for phage genome analysis (Besemer and Borodovsky, 1999; Delcher et al., 1999; Mills et al., 2003), the GeneMarkS self-training version automatically excludes candidate ORFs which are shorter than 300 bp, and assign ORFs from the 5′-most ATG codon (Besemer et al., 2001). These automated rules likely resulted in slight underestimation of gene numbers and coding density in phage genomes.

The program ARAGORN (Laslett and Canback, 2004) was used to detect tRNA and tmRNA genes in phage genome sequences.

#### Genome Mapping and Assigning Open Reading Frame (ORF) Families

The open-source program Phamerator (Cresawn et al., 2011) was used to map phage genomes in linear illustration and identify similarity between sequences. Predicted ORFs were assigned into families also using the program with a ClustalW threshold of 35% amino acid identity and a BLASTP score of 1e-50. Conserved protein domains in ORFs were identified by searching for hits from the NCBI Conserved Domain Database (CDD). To probe into the possibility of ‘ORFans’ (i.e., ORFs that shared no detectable relationships with other ORFs in this analysis) having homologs in undocumented phages, we compared the protein sequences of the 2,992 ORFans in our 130 genomes with the globally sampled virome dataset constructed by Paez-Espino and colleagues (Paez-Espino et al., 2016) using TBLASTN (Camacho et al., 2009). All the 125,842 viral genomes/contigs assembled from this large-scale metagenomic dataset were downloaded from the public FTP site http://portal.nersc.gov/dna/microbial/prokpubs/EarthVirome_DP/.

#### Genome Clustering

Genome alignment and calculation of percentage of nucleotide identity were performed with Kalign (Lassmann and Sonnhammer, 2005). A dot plot of whole genome comparison for all 130 phage DNA sequences was generated in Gepard 1.40 (Krumsiek et al., 2007) with a sliding window of 10 nucleotides, allowing for the visualization of pairwise similarity between genomes and assigning preliminary clusters. Phage genomes were incorporated into a cluster if they shared more than 45% nucleotide identity with the cluster’s genome members and there was nucleotide sequence similarity visually recognizable in the dot plot. Previous studies utilized the threshold 50% nucleotide identity (Hatfull et al., 2010; Pope et al., 2011), however, this value was applied for phages isolated from a single host (*Mycobacterium smegmatis*), while our study included phages infecting bacteria across the genus *Pseudomonas*, which potentially lead to greater variation in genome sequences. Therefore, we lowered the threshold parameter in our analysis to 45%. A second criterion for assigning members into cluster was that they share over 20 predicted protein families, to ensure detectable relatively conserved regions and synteny. Third, clusters were required to have at least three members. Phage genomes not meeting these three criteria were not included in clusters, identified as ‘singletons.’

Within each cluster, the variation of host bacterial species among phage members was investigated. Information of original host species was extracted from the ‘host’ field of the phage GenBank entry. To confirm the potential of phages, especially members of clusters with multiple hosts to infect different *Pseudomonas* species, we looked for signatures of past phage-host infections using the Clustered Regularly Interspaced Short Palindromic Repeats (CRISPR)/Cas (CRISPR-associated) spacer approach. All available spacer sequences identified in *Pseudomonas* bacteria were downloaded from the public database CRISPRdb (Grissa et al., 2007) and compared with phage genomes individually using BLASTN. Since spacer sequences, which range from 25 to 75 bp, are shorter than usual BLASTN query size, we utilized the following BLASTN parameters: maximum e-value of 0.3, word size 7, gap extension penalty 2, gap opening penalty 10, mismatch penalty 1, dust filtering off [adapted with modification from Edwards et al. (2016)].

#### Evolutionary Analysis

To investigate the genetic variation and pattern of evolution in predicted coding regions, we calculated the nucleotide diversity within selected ORF families (π_N_/π_S_). Since evolutionary analysis using π_N_/π_S_ test requires high confident alignment regions of ORF families, we increased ClustalW threshold value to 50% to ensure less diversity in ORF members and facilitate better alignments. All ORF families which have ClustalW score of at least 50%, BLASTP score threshold of 1e-50 and have 15 members or more were included in evolutionary analysis. Predicted protein-coding ORF sequences of each family were aligned using the MUSCLE program in MEGA7 software (Kumar et al., 2016). π_N_ and π_S_ were calculated individually for each ORF family using the software DNAsp v5.10.1 (Librado and Rozas, 2009). The π_N_ and π_S_ values of the ORF families were compared using the non-parametric Wilcoxon signed-rank test for paired data.

## Results

### Patterns of Genomic Variation

**FIGURE 1:**
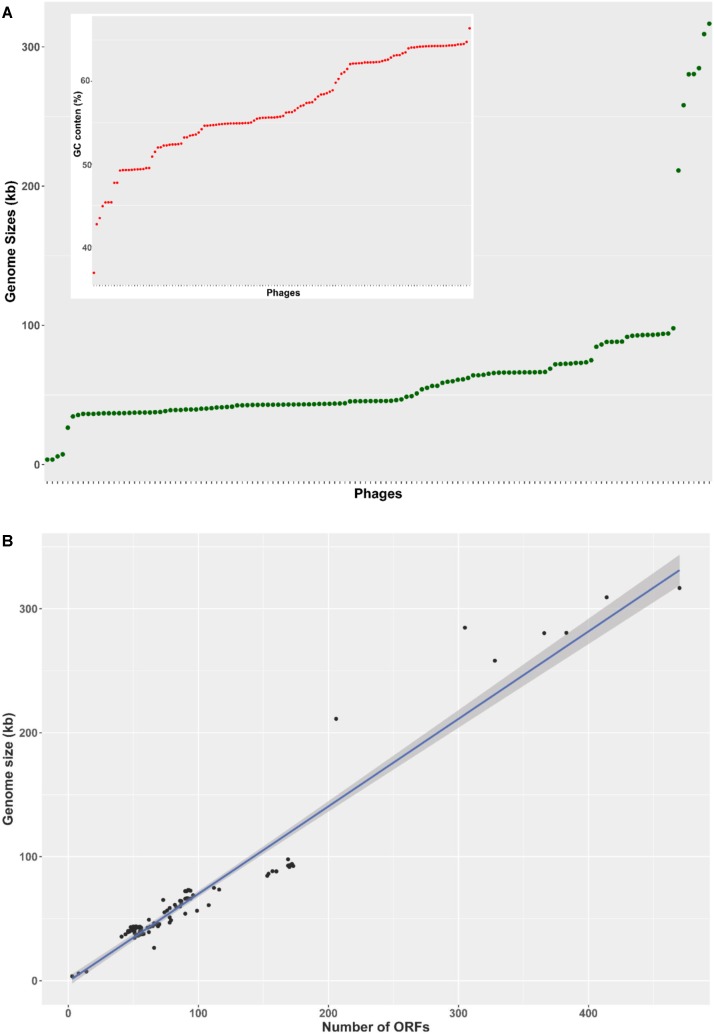
Genome characteristics of 130 *Pseudomonas* phages. **(A)** A rank-ordered plot of genomes sizes reveals a range of 3–316 kb and only a few genomes larger than 100 kb. Phages were rank-ordered on the *X* axis based on the property identified on the *Y* axis. **(B)** The number of predicted ORFs in phage genomes showed a strong, statistically significant correlation with genome size (*R*^2^ = 0.936, *p* < 0.001). The shading denoted 95% confident interval of the linear correlation (C). A ranked plot of G + C content reveals a range of 37–66%.

The basic genome metrics of the 130 phage sequences included in this study (host, genome size, G + C content, number of predicted ORFs, number of tRNA or tmRNA genes) were provided in **Supplementary Table S1**. The analyzed *Pseudomonas* phages showed broad diversity of genome size from 3 to 316 kb. The majority of phages (119 out of 130 - 92%) had genome size in the range from 35 to 100 kb, distributing uniformly across this interval. Four outliers were very small at less than 8 kb, and seven were larger than 200 kb (**Figure 1A**). This pattern was mirrored in the number of predicted ORFs (**Figure 1B**), as the four phages with the smallest genomes contained only 3–8 predicted ORFs, whereas the seven unusually large genomes harbored more than 200 putative ORFs. The remaining 119 phages encoded 41–173 ORFs. The overall average of putative ORFs per genome was 93.4. G + C content also varied greatly (ranging from 36.8 to 66.4%), averaging at 56.8% (**Figure 1A**, inset).

### Categorization of Phage Genomes Into Clusters

**FIGURE 2:**
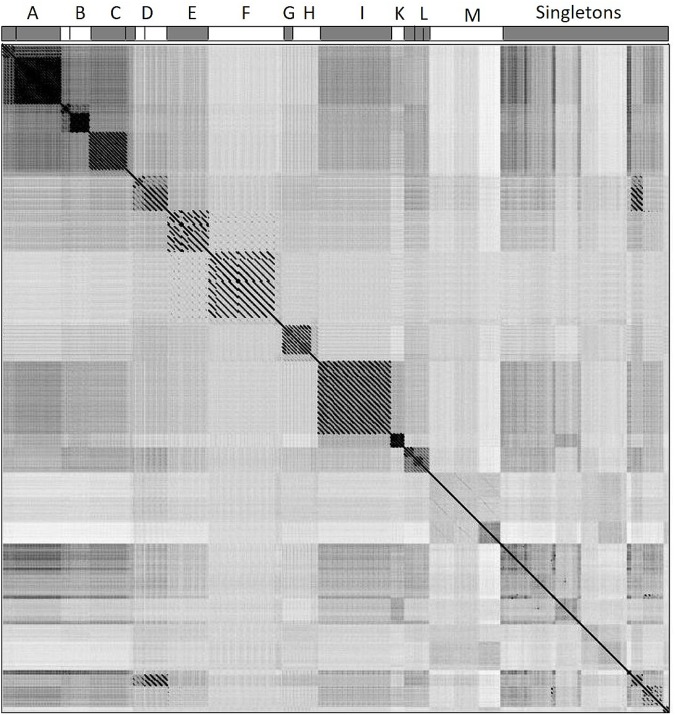
Whole-genome dot plot comparison of phage nucleotide sequences. All 130 genomes were concatenated into a single sequence, then plotted against itself with a sliding window of 10 bp and visualized by Gepard 1.40. 111 phage genomes were assigned to 12 clusters (A–M) and 30 phage genomes remained singletons. The assignment of phages to clusters A–M is shown at the top horizontal axis.

To assign phage genomes to clusters, we calculated pairwise sequence similarity between all possible pairwise phage genomes and performed whole genome dot plot analysis. The methods of clustering phages based on dot plot matrix were similar to those previously applied to mycobacteriophages (Hatfull et al., 2010; Pope et al., 2011). All 130 nucleotide genomes were concatenated into a single sequence and duplicated to form two axes, generating a dot plot matrix (**Figure 2**). If two sequences had high similarity, a diagonal would show at that location on the plot (the center diagonal line demonstrated the 100% similarity where sequences were compared to itself). The resulting dot plot matrix revealed 12 clusters (Clusters A through M) of phages sharing at least 45% nucleotide similarity; 30 genomes were not assigned to any cluster and remained singletons. Pairwise nucleotide similarity between phages of each cluster were reported in **Supplementary Table S2**. The number of members and pairwise nucleotide similarity between phage genomes of each cluster varied from cluster to cluster, e.g., cluster L has 8 members sharing at least 58% nucleotide similarity, while this number in cluster K is 75% among the 4 members (**Supplementary Table S2**). Some clusters (Clusters A through D, H, and L) were further divided into subclusters. Details of phage assignment to clusters and subclusters were shown in **Supplementary Table S1**. Basic characteristics of members in each cluster including morphotype (according to ICTV classification system), host species from which the members were isolated, average genome size, number of ORFs, GC content, number of tRNAs were also provided in **Supplementary Table S1**.

### Characterization of Open Reading Frames (ORFs) in Phage Genomes

**FIGURE 3:**
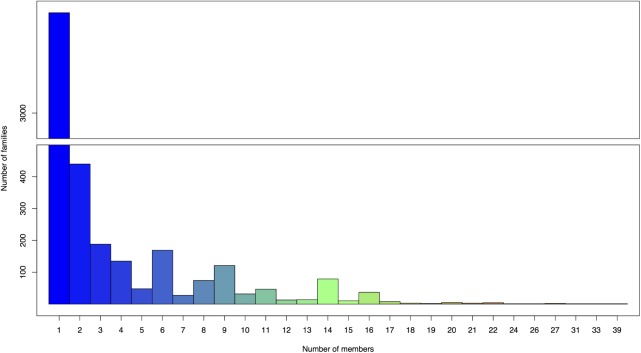
Number of members in ORFs families assigned by Phamerator. The largest family has 39 members and 2,992 families (67.1% of the total 4,462 ORF families generated) only have one member.

The annotation process predicted a total of 12,139 putative ORFs ranging from 54 bp to 12 kb in size, with an average length of 650 bp among the 130 genomes analyzed. The predicted ORFs were assigned to groups of closely related sequences (ORF families) using Phamerator with a ClustalW threshold of 35% amino acid identity and a BLASTP score of 1e-50. In genomic maps, putative ORFs were colored and numbered according to Phamerator assigned ORF family (white color denoted ORFs having no similarity with other ORFs, equal to the threshold BLASTP 1e-50 or smaller). A total of 4,462 ORF families were generated with an overall average number of only 2.72 members. The largest family had 39 members (family 107) and 2,992 families (67.1% of the total) only had one member (**Figure 3**). Out of these 2,992 ORFans, 365 sequences (12.2%) had significant hits (maximum *e*-value set at 1e-50) with the contigs in Paez-Espino et al. (2016) virome dataset, and with a more relaxed threshold (*e*-value 1e-25), homologs of 665 ORFans (22.22%) were found.

Open reading frame density of all phage genomes varied from 0.83 to 2.50 per kb, averaging at 1.42 ORFs per kb. This value was highly similar between members within each cluster, i.e., shown in very small deviations from the mean (**Table 1**). Members of Cluster E and F showed higher mean ORF densities among all clusters, ranging from 1.73 to 1.87 ORFs per kb. However, the highest average numbers of ORFs each kb were observed in two singletons Pf1 and phi_Pto-bp6g (1.91 and 2.49). The lowest numbers of ORFs per kb (0.83 and 0.84) were found in PP7 and PRR1, the only two Leviphages included in this study.

**Table 1:** Summary of phage characteristics by clusters.

Cluster	# of members	ICTV Family	Host(s)	# ORFs	# ORFs per kb	Genome size (bp)	GC content	# of tRNAs
A	20	Siphoviridae	*P. aeruginosa*	53.6 ± 2.0	1.43 ± 0.06	37289.5 ± 1174.0	64.0 ± 0.5	0 ± 0
B	6	Siphoviridae	*P. aeruginosa*	82.0 ± 4.3	1.37 ± 0.04	59645.3 ± 1973.1	64.2 ± 0.4	0 ± 0
C	13	Podoviridae	*P. aeruginosa, P. fluorescens*	52.0 ± 2.4	1.21 ± 0.06	42945.2 ± 522.7	62.0 ± 1.0	0 ± 0
D	6	Podoviridae	*P. aeruginosa*	99.5 ± 11.4	1.36 ± 0.14	73158.5 ± 979.3	54.4 ± 0.8	0 ± 0
E	6	Myoviridae	*P. aeruginosa*	157.3 ± 3.2	1.80 ± 0.02	87273.0 ± 1534.8	54.7 ± 0.1	3 ± 0
F	10	Myoviridae	*P. aeruginosa, P. syringae*	171.1 ± 1.4	1.83 ± 0.04	93570.7 ± 1668.2	49.2 ± 0.5	15 ± 1.8
G	3	Podoviridae	*P. aeruginosa*	68.3 ± 2.9	1.52 ± 0.03	45068.7 ± 903.5	52.2 ± 0.2	3.3 ± 0.5
H	7	Podoviridae	*P. aeruginosa, P. fluorescens, P. putida*	68.0 ± 1.4	1.49 ± 0.03	45698.1 ± 291.3	52.3 ± 0.5	2.14 ± 1.5
I	14	Myoviridae	*P. aeruginosa*	91.5 ± 2.7	1.38 ± 0.02	66104.4 ± 1066.5	55.4 ± 0.3	0 ± 0
K	4	Siphoviridae	*P. aeruginosa*	54.3 ± 1.3	1.26 ± 0.03	43031.0 ± 157.3	53.9 ± 0.5	0.25 ± 0.5
L	8	Podoviridae	*P. aeruginosa, P. fluorescens, P. putida, P. plecoglossicida, P. chlororaphis*	48.1 ± 2.5	1.20 ± 0.03	40032.5 ± 1325.6	57.0 ± 0.7	0 ± 0
M	3	Myoviridae	*P. aeruginosa, P. chlororaphis*	416.7 ± 52.1	1.38 ± 0.09	302072.0 ± 19192.2	43.3 ± 5.7	6.3 ± 3.1

**FIGURE 4:**
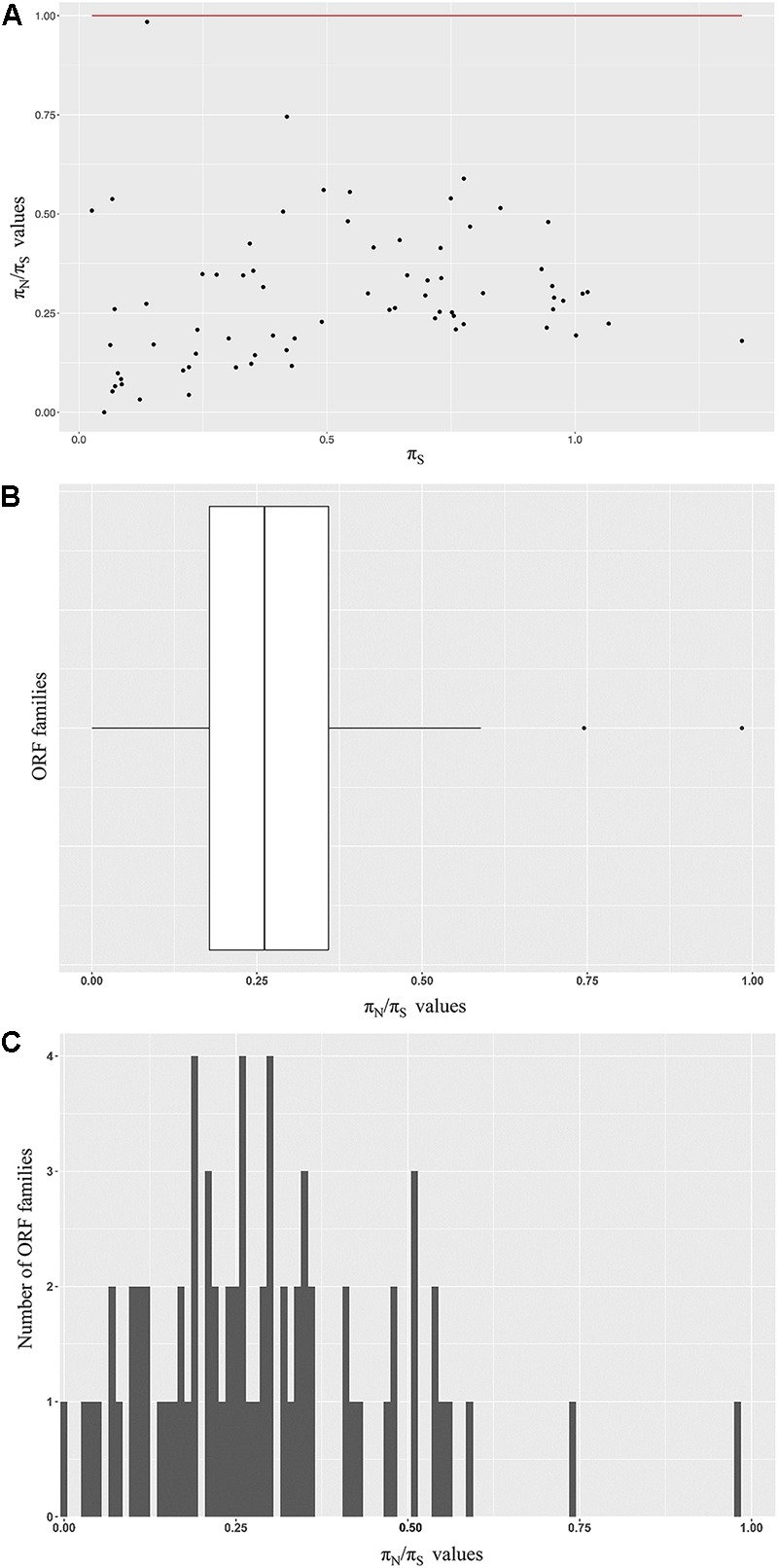
Modes of selection acting on a subset of ORF families. **(A)** Seventy-two families were chosen for further evolutionary analysis. The π_N_/π_S_ ratio of each family are shown on the *Y* axis. The *P*s values are shown on the *X* axis. The red line indicates the π_N_/π_S_ = 1. **(B)** Distribution of the π_N_/π_S_ ratio of the ORF families analyzed. **(C)** A histogram of the π_N_/π_S_ values among ORF families analyzed. The majority (62/72 – 86%) of the families included has a π_N_/π_S_ ratio ranging from 0 to 0.5.

To investigate the mode of selection acting on phage ORFs, we calculated the nucleotide diversity at non-synonymous sites and compared it to the diversity at synonymous sites (the π_N_/π_S_ ratio) for a select set of 72 ORF families. π_N_/π_S_ at or near one suggested that the ORF sequences of interest were likely evolving under neutrality; π_N_/π_S_ values greater than one implied positive selection and values less than one indicated purifying selection. We observed a broad pattern of π_N_/π_S_ values distribution, however, all ORF families analyzed had π_N_/π_S_ value under 1 (**Figure 4A**). The majority of the families had π_N_/π_S_ ratio closer to zero (62/72 families – 86% have π_N_/π_S_ within the range from 0.0 to 0.5) (**Figures 4B,C**).

### Diversity of Phage Hosts Within Clusters

**FIGURE 5:**
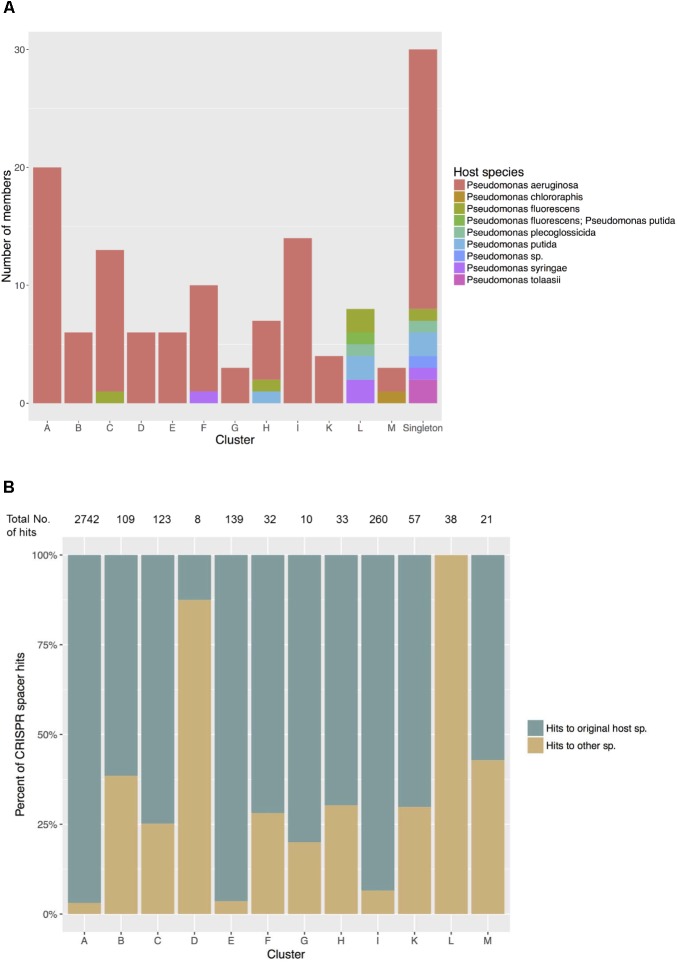
**(A)** Host species of phages in each cluster. Five clusters show closely related phages infecting different host species. **(B)** Matches between phage sequences in each cluster and CRISPR spacers in *Pseudomonas* host species. Significant matches were recorded as hits to spacers predicted in their original host species and hits to other *Pseudomonas* species. The total numbers of hits, regardless of the types found in each cluster were shown at the top.

Of the 12 *Pseudomonas* phage clusters defined here, five contained phage members isolated from more than one host species (Clusters C, F, H, L and M) (**Figure 5A**). On the extreme end, the eight members of Cluster L were isolated from five different hosts: *P. putida, P. fluorescens, P. tolaasii, P. syringae*, and *P. plecoglossicida*, while all members shared at least 58% nucleotide similarity with others in cluster L (**Supplementary Table S2**). When compared with the *Pseudomonas* bacterial CRISPR spacer library, all these phages showed matches with spacers of multiple *Pseudomonas* species that differed from the original hosts. Meanwhile, in clusters of phages all isolated from one sole species, especially Clusters A, E and I (all originated from *P. aeruginosa*), members tended to share more sequence similarities with spacers of *P. aeruginosa* - their host species (**Figure 5B**).

**FIGURE 6:**
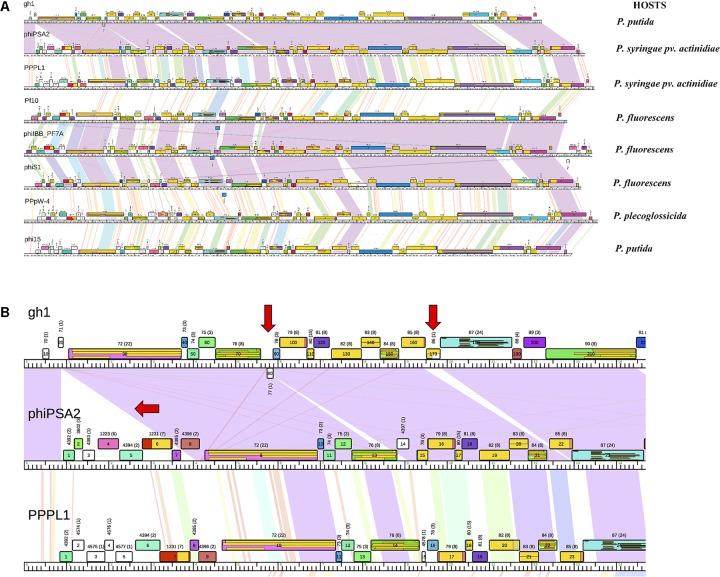
**(A)** Genomic map of phages in Cluster L. Phage genomes were mapped using Phamerator. Genomes were arranged in the map according to the assigned subclusters: subcluster L1 with gh-1, phiPSA2, PPPL-1, subcluster L2 with Pf-10, phi-S1, phiIBB-PF7A, and subcluster L3 with phi15 and PPpW-4. Boxes indicate predicted ORFs, numbers and colors are assigned according to predicted protein families. White boxes denote ORFs that have no similarity at an *E*-value 1e-50 or smaller to other predicted ORFs. Shading between genomes indicates regions of pairwise nucleotide similarity and was coded in color spectrum so that color indicates nucleotide similarity (violet representing highest similarity with an E-value of zero and red being similarity with *E*-value of 1e-50). **(B)** Close-up view of subcluster L1 map. Red arrows indicate breaks of synteny. Yellow boxes within ORFs display conserved domain hits from CDD database, separated by lines if there are multiple hits found in one ORF.

Genomic map of phages in Cluster L was shown in **Figure 6A**. Within subcluster L1 and L2, we observed long regions of violet shading indicating long conserved regions between phage genomes. Meanwhile, between subclusters, this relationship was apparently weaker with shading toward the red end of the color spectrum. Regions of high similarity and same-colored ORF blocks shown on the map indicated prevalent synteny. Breaks in synteny were also evident as interspersed white blocks and little or no sequence similarity between genome sequences. An example of such synteny breaks was shown in **Figure 6B**. Between the two phage genomes gh-1 and phiPSA2 of subcluster L1, the presence of gh-1 ORFs (gh1_80 and gh1_170) had interrupted the synteny organization.

On mapped sequences, conserved domain hits from CDD database could be found, including not only hits from *Pseudomonas* bacteria and their phages, but also from different prokaryotes and viruses. In many ORFs, multiple overlapped hits corresponding to the same portion of the ORF were observed. This pattern was more likely to be found in ORFs belonging to large families (e.g., gh1_30, gh1_150, phiPSA2_9, phiPSA2_13, **Figure 6B**). The domains identified usually involves in conserved phage functions (e.g., ORF gh1_150 contains peptidoglycan recognition protein domain, T3-like lysozyme domain, and domain of *N*-acetyl-anhydromuramyl-L-alanine amidase, which cleaves the amide bonds between *N*-acetyl-anhydromuramyl and L-amino acids in bacterial cell wall).

## Discussion

### Variation in *Pseudomonas* Phage Genomes and Genes

The 130 phage genomes analyzed show a wide variety of G + C content, from as low as 37–66%. Interestingly, since the average G + C percentage of bacterial *Pseudomonas* sp. genomes is in the range from 58 to 66% (Winsor et al., 2016), the G + C content of a large number of *Pseudomonas* phage genomes in this study is much lower than that of its host (**Table 2**). Similar observation was noted in a sample of 18 *P. aeruginosa* phages (Kwan et al., 2006), and did not agree with other findings that phage G + C content is usually similar to that of the host (see examples in **Table 2**). The low G + C percentage in *Pseudomonas* phage genomes may indicate an active history of shifting from other bacterial hosts where the phages acquired low G + C content sequences via horizontal gene transfer (HGT).

**Table 2:** Examples of G + C content in different phages and their bacterial host.

Phage	G + C content of phage %	G + C content of host
phiKZ	36.83	*P. aeruginosa* strain PA01 (66.6%) (Labaer et al., 2004)
phiPA3	47.73	
PA2	54.86	
PABG	55.82	
UFV-P2	51.47	*P. fluorescens* (60 to 66%) (Cornelissen et al., 2012)
phi_Pto-bp6g	42.71	*P. tolaassii* strain 6264 (60.6%) (Winsor et al., 2016)
eiAU	55.37	*Edwardsiella ictaluri* (57%) (Carrias et al., 2011)
eiDWF	55.54	
eiMSLS	55.77	
*Staphylococcus aureus* phages	33.7	*Staphylococcus aureus* (32.9%) (Kwan et al., 2005)
mycobacteriophages	63.4	*Mycobacterium smegmatis* (67.4%) (Mohan et al., 2015)
*Streptococcus pneumoniae* phages	39.8	*Streptococcus pneumoniae* (39.7%) (Kwan et al., 2006)

*Many of the Pseudomonas phages included in this study have notably lower G + C content than their host, in contrast with the relative similarity between phages of other bacteria.*

Cluster assignment based on sequence similarity and homologs is supported by highly similar properties, i.e., low standard deviation of the within-cluster average, such as morphotype, genome size, GC percentage, number of predicted ORFs (**Table 1**). The proportion of singletons (30/130 phages – 23.1%) is notably higher compared to previous results of other comparative genomic studies: 1.3% of 627 mycobacteriophage genomes (Pope et al., 2015); 5.3% of 337 *Enterobacteriaceae* phages (Grose and Casjens, 2014), and 18.1% of 83 *Bacillus* phages (Grose et al., 2014) were designated to be singletons. It is worth noting that the small percentage of singletons in mycobacteriophage might be due to the very large scale of survey on phages isolated from a single species (*Mycobacterium smegmatis*). As the survey broadened to higher taxonomic level yet smaller sample size, the percentage of singletons increased noticeably. The large number of singletons suggests that the current stage of discovery has revealed just part of the dynamic diversity in the world of *Pseudomonas* phages. Adding new genomes could bring a singleton into a cluster by identifying intermediate phage relatives, therefore further sampling, profiling and reassigning of clusters might be necessary to better evaluate the extent of *Pseudomonas* phage diversity.

Genome mosaicism was observed extensively in all phage genomes with the remarkable frequency of ORF modules and breaks in synteny between genomes. ORFs of the same family consistently located between different flanking ORFs. This pattern of pervasive mosaicism is well in line with previous findings in *Enterobacteriaceae* phages, *S. aureus* phages and mycobacteriophages (Kwan et al., 2005; Pope et al., 2011; Grose and Casjens, 2014). The mosaicism may suggest (1) high activities of HGT and (2) phage evolution to drop unnecessary genes to keep the genome minimal and efficient as they adapt to new purposes. Dynamic HGT also hints at a flexible host range, which is needed for more opportunities to gain access to a larger gene reservoir. Other genomic events could also contribute to the large-scale mosaicism such as transposition (Edgell et al., 2010), cleavage by endonucleases (Kristensen et al., 2013), phage recombinases acting on relaxed homology (De Paepe et al., 2014), and mistakes in genome replication and host repair mechanism during the prophage phase.

The great diversity of *Pseudomonas* phage was also indicated on the scale of genes. With a ClustalW threshold of 35% amino acid identity and a BLASTP score of 1e-50, the largest ORF family has only 39 members, which is remarkably small compared to the largest family (104 members) that (Pope et al., 2011) assigned with similar thresholds from 80 mycobacteriophage genomes. Moreover, 67.1% of the predicted ORF families have only one member, while the numbers of one-member families identified in studies of mycobacteriophages and *Bacillus* phages are much lower at 47.2 and 58%, respectively. Remarkably, a large number of presumptive insertions/ deletions in genomes are unique ORFs (displayed in genome map as white boxes), and rarely contain known conserved domains. The vast number of unique ORFs and the diversity of ORF families suggest a large gene influx from novel bacterial hosts and/or other phages by HGT. Among the 2,992 ORFans identified, a considerable proportion (665 sequences – 22.2%) had significant TBLASTN hits with the global virome dataset despite a stringent threshold (1e-25). This result further demonstrates the largely unexplored gene reservoir of *Pseudomonas* phages, with many potential homologs with undocumented phage sequences in nature.

The number of predicted ORFs was significantly positively correlated with phage genome sizes (*R*^2^ = 0.936, *p* < 0.001 – **Figure 1B**) in our study. ORFs account for more than 80% of the total genome sequence space for all phages examined (**Supplementary Table S1**) with an average coding percentage of 92.4%, indicating their high genetic efficiency, which is consistent with observations of previous studies, e.g., of mycobacteriophages (Rohwer et al., 2014), *Staphylococcus aureus* phages (Kwan et al., 2005). Mean ORF density was at 1.42 ORFs per kb, which is slightly less than that in mycobacteriophages and *Staphylococcus aureus* phages (1.69 and 1.67 genes per kb, respectively) (Kwan et al., 2005; Hatfull et al., 2010). The only two Leviphages included in this study, PP7 and PRR1, have the lowest ORF densities (0.83 and 0.84 ORFs per kb, respectively), while possessing the smallest genomes among the 130 phages (3,588 and 3,573 kb). This may initially seem counter-intuitive, since small phages must compress a minimum number of genes required for surviving in very small sequence space, which should result in higher number of ORFs per kb – for example, Microphage φX174 accommodates 11 genes over the length of only 5,386 bp through gene overlap involving multiple reading frames (Rohwer et al., 2014). However, small phages also must maintain minimum sizes and extra nucleotide sequences to allow for efficient packaging, thus decrease their ORF density. This was observed in phage lambda sequence, where non-coding sites (*cos*) are essential for DNA cleavage, processing, duplex nicking, enzyme binding (Catalano et al., 1995; Feiss and Catalano, 2013). It is possible that non-ORF sequences in these small *Pseudomonas* phage genomes perform important functions that we do not yet understand.

### Insights Into Phage Host Range

Eight members of Cluster L were reported to have five different host species while sharing at least 58.7% nucleotide identity. While these five hosts appear to be more related to each other than to *P. aeruginosa* (Ait Tayeb et al., 2005), interestingly, member of Cluster M, isolated from *P. aeruginosa* and *P. chlororaphis*, also share as high as at least 50.9% identity. Although it was expected that percentage of identity among Cluster M is smaller than that of clusters with members isolated from the same host species (e.g., members of Cluster K, all isolated from *P. aeruginosa* - different strains – share at least 75.2% identity), this sequence clustering is remarkable as *P. aeruginosa* and *P. chlororaphis* are much further apart in the *Pseudomonas* phylogenetic tree (Ait Tayeb et al., 2005). This pattern of clustering suggests a flexible host range not only among strains within one species, but also could expand to between species. The dynamics would allow for better adaption to their fast-changing bacterial hosts.

To further evaluate phage potential for broad host ranges beyond a single bacterial species, we computationally investigated the history of past infections in all *Pseudomonas* species by comparing each phage genome with all available CRISPR spacers originating from *Pseudomonas* bacterial genomes. CRISPR modules in the genome provide bacteria with an adaptive immunity against viruses and mobile genetic elements (Horvath and Barrangou, 2010). CRISPR arrays consist of interspaced repeated sequences that are separated by short different sequences named spacers. These fragments may represent a part of phage sequences inserted into CRISPR arrays on bacterial genome during previous infections and are constantly replaced and heritable. Therefore, spacers provide a paleogenomic window into recent phage infections. Members of Cluster L showed matches to various species, 100% of which are different from their host and might suggest the ability and/or a history of attacking different species in *Pseudomonas*, and then shifting to the current host, while in *P. aeruginosa* phages of Cluster A, E, and I, this variation is much less extensive (**Figure 5B**). We note that Cluster D, also consists of all *P. aeruginosa* phages, shows a lower percentage of matches to *P. aeruginosa* itself (12.5% – **Figure 5B**), however, it is also worth noting that the total number of hits is only eight, hence this low proportion might be a result of error sampling. While the spacer library in *P. aeruginosa* is expected to be better documented than that in other *Pseudomonas* species, which could in part explain the dominant proportion of *P. aeruginosa* hits of phages in Cluster A, E, and I, and the lack of matches to the recorded hosts of all phages in Cluster L, the presence of similarities to species other than the original hosts does imply the capability of host shifting.

### Purifying Selection Is Prevalent Among a Subset of Predicted ORF Families Analyzed

Nucleotide diversity at non-synonymous and synonymous sites among multi sequences, π_N_/π_S_, and Ka/Ks, which performs pairwise comparisons in different species, have been considered useful tools to evaluate the type of natural selection acting on coding regions (Howe and Denver, 2008; Zhang et al., 2016; Chen et al., 2017). The ratio Ka/Ks is frequently used in well-defined species, calculated by pairwise comparisons and highly time dependent (Rocha et al., 2006). Since we were comparing the diversity between ORF orthologs in multiple phages, of which the species taxonomic classification is not well established (Lawrence et al., 2002), we performed the π_N_/π_S_ test to evaluate the selection pressure on phage putative genes.

We found that all of the 72 ORF families included have a π_N_/π_S_ ratio less than 1.0, which implies a reduced diversity in non-synonymous sites and a history of long term purifying selection (Wilcoxon signed-rank test, *p* = 8.5e-14). Eighty-six percent (62/72) families have π_N_/π_S_ ratio closer to zero, pointing to relatively strong purifying selection. Out of ten families indicating weaker purifying selection (π_N_/π_S_ > 0.5), six showed π_S_ < 0.5. As lower values of π_S_ points to smaller possibility of saturation at synonymous sites, these six families provided the most reliable evidence for purifying selection. Two families were annotated as housekeeping genes with structural function, i.e., major capsid protein (family 1310) and putative large terminase subunit (family 5602). The other four families were unannotated and have no known domains identified. No signal of positive selection, which favors synonymous substitutions and results in π_N_/π_S_ ratios above 1.0, was observed. This pattern agrees with previous studies of mycobacteriophages and cyanophages. Weigele et al. (2007) measured the ratio Ka/Ks for all pairwise orthologs in 20 mycobacteriophages and 5 cyanophages and found that the values average at about 0.25 and 0.01, respectively. Based on the fact that the majority of protein coding genes have π_N_/π_S_ < 1, the ratio offers the potential to assist the identification of true genes (Nekrutenko et al., 2002). Although the results of π_N_/π_S_ and similar genetic code-based tests alone cannot absolutely confirm the accuracy of gene calling, they offer informative complimentary strategies to evaluate the veracity of candidate ORFs identified by annotation tools and can be applied to any putative coding sequences.

The genomic data of 130 phages included in this study has revealed extensive gene diversity and strong purifying selection acting on genes of *Pseudomonas* phages. Nevertheless, we note that the dataset used for the present study was downloaded in February 2016, incorporating the 130 complete *Pseudomonas* phage sequences then available on the NCBI GenBank database. In the time it took to execute the present analyses and write-up, the number of available genomes has more than tripled (391 *Pseudomonas* phage genomes available in May 2018). Exhausting all available genome entries in public databases proves to be a continual problem for comparative genomic analyses, especially in this instance, as phages with small genomes are constantly added to databases at accelerating rates. However, given the high genetic diversity, cluster structures, and the abundance of unique genes with no apparent relatives, future analysis including an increased number of sequences will provide more information about the genetic diversity and evolution of the world of *Pseudomonas* phages. Expanding the scope of analysis with the ever-increasing numbers of genomes is expected to decrease the number of ORFans as newly identified homologs in the broader gene reservoir could bring them into families; though how many genomes will be required to see substantial ORFan number reductions remains unclear.

Currently, the number of complete *Pseudomonas* phage genomes available from GenBank is heavily skewed toward *P. aeruginosa* phages. In this study, 107 out of the 130 phages analyzed were listed as isolated from *P. aeruginosa. P. aeruginosa* is a major human pathogen with the increasing ability to develop antimicrobial resistance and the potential for phage therapeutic strategy against *P. aeruginosa* infections has long been recognized. Consequently, it was anticipated that *P. aeruginosa* bacteriophages would receive preferential attention, and thus would be more frequently profiled and investigated. To achieve a more comprehensive understanding of phages infecting *Pseudomonas*, future research should include more sequences of phages isolated from other host species.

## Author Contributions

AH and DD designed the study, contributed to revision, read and approved the final manuscript. AH organized the data, performed the data analysis, and drafted the manuscript. DD conceived the study and critically revised the manuscript.

## Conflict of Interest Statement

The authors declare that the research was conducted in the absence of any commercial or financial relationships that could be construed as a potential conflict of interest.

## Supplementary Material

TABLE S1 Basic genome metrics of the 130 *Pseudomonas* phage sequences included.

TABLE S2 Pairwise nucleotide similarity between phages of each cluster.
